# Consumer perception data and scientific arguments about food packaging functionalities for fresh strawberries

**DOI:** 10.1016/j.dib.2018.09.034

**Published:** 2018-09-15

**Authors:** Bruno Yun, Patrice Buche, Pierre Bisquert, Sandrine Costa, Madalina Croitoru, Julien Cufi, Valérie Guillard, Alrick Oudot, Rallou Thomopoulos

**Affiliations:** IATE-LIRMM-MOISA, University of Montpellier - INRA, 2 place Pierre Viala, F-34060 Montpellier Cedex, France

## Abstract

This data article contains data characterizing consumer perception and scientific arguments about food packaging functionalities for fresh strawberries. These data are associated with the article “Choice of environment-friendly food packagings through argumentation systems and preferences” (see Yun et al., 2018). These data are stored in a public repository structured by an ontology. These data could be retrieved through the @Web tool, user-friendly interface to capitalize and query data (Buche et al., 2013; Guillard et al., 2017). The @Web tool is accessible online at http://pfl.grignon.inra.fr/atWeb/.

**Specifications table**

**Table:** 

Subject area	*Consumer perception*
More specific subject area	*Food packaging functionalities*
Type of data	*Table*
How data was acquired	*A survey upon a sample of 845 people, representative of the French population in terms of age and socio-professional categories.*
	*Arguments expressed by a food packaging scientific expert.*
Data format	*Raw and analyzed.*
Experimental factors	*Transformation of consumers’ answers to poll into arguments is defined in the related research article*
Experimental features	*Transformation of consumers’ answers to poll into arguments is based on a majority vote.*
Data source location	*University of Montpellier, FR-34060, France*
Data accessibility	*Data are accessible in a public repository*
Related research article	*Ranking semantics for the choice of environment-friendly food packagings (submitted to Environmental Informatics)*

**Value of the data**

• A unique set of consumer perception data and scientific expert arguments indispensable in food engineering to design relevant food packaging for fresh foods.
• These data could be used to rank food packaging solutions according to consumer perception and expert knowledge.
• These data could serve as benchmark for other researchers coping with research on argumentation and multi-criteria decision support system.

## 1 Data

Consumer perception data are extracted from a survey upon a sample of 845 people representative of the French population in terms of age and socio-professional categories. Food packaging expert arguments have been registered during meetings of the INRA-CIRAD GloFood Pack4Fresh project. These data are stored in a data warehouse called @Web (https://www6.inra.fr/cati-icat-atweb/) in which the data management is guided by ontology (http://pfl.grignon.inra.fr/atWeb/ and [2], [3]).

**Table:** 

Data type	Table DOI^a^	Amount of data
**Consumers’ answers to poll**		
Consumers’ answers to poll for Wood packaging	http://doi.org/10.15454/GNBUFH	72
Consumers’ answers to poll for Plastic with plastic film	http://doi.org/10.15454/UBPOQG	90
Consumers’ answers to poll for Plastic rigid lid	http://doi.org/10.15454/HKIQFJ	72
Consumers’ answers to poll for Plastic not closed	http://doi.org/10.15454/IHVM9P	72
**Arguments generated from consumers’ answers**		
Wood packaging consumers’ arguments	http://doi.org/10.15454/F4C8I0	9
Plastic with plastic film consumers’ arguments	http://doi.org/10.15454/GMWB8Q	11
Plastic rigid lid consumers’ arguments	http://doi.org/10.15454/DC9PYL	9
Plastic not closed consumers’ arguments	http://doi.org/10.15454/NM3WET	9
**Food packaging expert arguments for all packaging solutions**	http://doi.org/10.15454/VDNRH6	9

a Access to data table is provided using the DOI metadata “Link to data”.

## 2 Experimental design, materials, and methods

Consumers’ answers to poll are extracted from a survey upon a sample of 845 people representative of the French population in terms of age and socio-professional categories.

Description of the sample

**Table:** 

Total sample	Number	%
	845	100
Female	501	60,4
Male	344	39,6
20–34 years old	208	24,6
35–49 years old	230	27,2
50–64 years old	218	25,8
65 years old and +	189	22,4
High CSP	235	27,8
Low CSP	238	23,8
inactive	374	37,4

• High CSP: farmers, entrepreneurs, artisan, manager, retailer, businessmen, intellectual works
• Low CSP: employees, workers
• Inactive: unemployed, students, retired persons

A set of 12 questions corresponding to different criteria has been asked for each of the 4 packaging alternatives: Wood packaging, Plastic with plastic film, Plastic rigid lid, Plastic not closed. In the following, the example of question “Is Wood packaging harmful for strawberries?” involving the criterion “harmful” will be used to illustrate the transformation of consumers’ answers to poll into arguments.

Accepted answers to questions are:

• Not agree at all
• Rather disagree
• Neither agree nor disagree
• Somewhat agree
• Totally agree
• Don’t know

Transformation of consumers’ answers to poll into arguments, defined in [1], is based on a majority vote. The answers are aggregated into 3 groups:

• “Not agree at all” and “Rather disagree” are sum up in No group.
• “Totally agree” and “Somewhat agree” are sum up in Yes group
• “Neither agree nor disagree” and “Don’t know” are sum up in Neutral group

For each question, if cardinality of Neutral group is strictly superior to the sum of the cardinalities of Yes and No groups, then no argument can be generated,

Else if cardinality of No group is strictly inferior to cardinality of Yes group then argument “Wood packaging is harmful for strawberries” is generated,

Else then argument “Wood packaging is not harmful for strawberries” is generated.

Food packaging expert arguments have been expressed after reading the arguments generated from consumers’ answers.
