# Supplementary material for: Consumer perception data and scientific arguments about food packaging functionalities for fresh strawberries
Source: Data Brief. 2018 Sep 15;20:1924–7. doi: 10.1016/j.dib.2018.09.034 (PMC6171093; doi:10.1016/j.dib.2018.09.034)
Supplement: Supplementary file 1 — Supplementary material [file mmc1.pdf]

Manuscript No.: DIB-D-18-01905

Conflict of interest form

'Consumer perception data and scientific arguments about food packaging functionalities for fresh strawberries'

We are no conflict of interest to declare.

With best regards,

Montpellier, the 4th of September, 2018

Patrice Buche
Research engineer with INRA

Institut National de la Recherche Agronomique
Etablissement public à caractère scientifique et technologique placé sous la tutelle conjointe des ministres chargés de la recherche et de l'agriculture
UMR IATE – 2, place Pierre Viala – 34060 Montpellier cedex 02 FRANCE
Tél. : +33499612283 – Mail : buche@supagro.inra.fr
http://umr-iate.cirad.fr/axes-de-recherche/ingenierie-des-connaissances/membres/patrice-buche-s-cv
